# Biochemical description of ozonized propolis and its activity against pathogenic microorganisms, cancer cells, and inflammation with molecular docking interaction

**DOI:** 10.1371/journal.pone.0332224

**Published:** 2025-09-17

**Authors:** Aisha M. H. Al-Rajhi, Sulaiman A. Alsalamah, Husam Qanash, Abdulrahman S. Bazaid, Abdu Aldarhami, Hashim R. Felemban, Hattan S. Gattan, Norah Mohammed AlZamil, Dalia Daws, Tarek M. Abdelghany

**Affiliations:** 1 Department of Biology, College of Science, Princess Nourah bint Abdulrahman University, Riyadh, Saudi Arabia; 2 Department of Biology, College of Science, Imam Mohammad Ibn Saud Islamic University (IMSIU), Riyadh, Saudi Arabia; 3 Department of Medical Laboratory Science, College of Applied Medical Sciences, University of Ha’il, Hail, Saudi Arabia; 4 Medical and Diagnostic Research Center, University of Ha’il, Hail, Saudi Arabia; 5 Department of Microbiology and Parasitology, Faculty of Medicine, Umm Al-Qura University, Al-Qunfudah, Saudi Arabia; 6 Department of Medical Laboratory Sciences, Faculty of Applied Medical Sciences, King Abdulaziz University, Jeddah, Saudi Arabia; 7 Special Infectious Agents Unit, King Fahad Medical Research Center, Jeddah, Saudi Arabia; 8 Department of family and Community medicine, College of Medicine, Princess Nourah bint Abdulrahman University, Riyadh, Saudi Arabia; 9 Pharmacy Department, Jazan University Hospital, Jazan University, Jazan, Saudi Arabia; 10 Botany and Microbiology Department, Faculty of Science, Al-Azhar University, Cairo, Egypt; Universidad San Francisco de Quito - Campus Cumbaya: Universidad San Francisco de Quito, ECUADOR

## Abstract

Numerous experimental investigations conducted on different natural compounds; however, their effectiveness remains insufficient for overcoming the health problems. The effect of ozone on phytochemical characterization of propolis with its biological activities was investigated currently. HPLC showed that ozonized propolis (OP) contains several compounds with high concentrations like hesperetin, rosmarinic acid, coumaric acid, caffeic acid, and chlorogenic acid compared to non-ozonized propolis (NOP). Inhibition zones, killing kinetic time, MID and MBD indicated the effective role of OP against *Salmonella typhi, Klebsiella pneumoniae, Bacillus subtilis,* and *Staphylococcus aureus* besides *Candida albicans* compared to NOP*.* Low IC_50_ value (9.91 ± 1.25 μg/mL) was attributed to OP while NOP provide IC_50_ value 26.05 ± 0.50 μg/mL as antioxidant agent via DPPH. Inhibition of protein denaturation as a marker of anti-inflammatory was recorded for OP with IC_50_ value of 6.46 ± 0.66 μg/mL compared to the IC_50_ value of NOP (11.32 ± 1.33 μg/mL). Caco-2 cells line was inhibited with morphological changes by either OP or NOP, however OP reflected excellent IC_50_ 9.9 ± 2.98 μg/mL contrast NOP (IC_50_ 41.43 ± 0.62 μg/mL). Flow cytometric analysis of Caco-2 cells documented the apoptosis caused by propolis particularly NOP. The current study adopts a computational method to investigate the possible antibacterial and anticancer properties of rosmarinic acid and hespertin as main constituents of propolis. Rosmarinic acid and hespertin were docked as ligands against *K. pneumonia* (PDB ID: 6T77) and Caco2 cells (PDB ID: 1M17) receptors.

## Introduction

Propolis is a gummy viscous material, which is harvested from bark and buds of trees. Because bees employ it to seal holes, cover surfaces, and fill holes in their hives, it is also recognized as “bee glue.” This creates a sterile habitat that shields the bees from microbes. It can be regarded to be a powerful weapon versus viruses, bacteria, and other harmful spores that may attack the colony of bee [1]. Chemical composition of propolis is considered a critical factor for determining its bioactivity. In current decade, there has been extensive interest in employing propolis in hospitals for repress microorganism’s development due to the rise in drug resistance. Argentinian propolis reflected strong antifungal potential versus a wide range of yeasts and molds, where the most sensitive were *Trichophyton mentagrophytes, Microsporum gypseum*, and *Trichophyton rubrum* [2]. *Rhizopus stolonifer*, *Fusarium oxysporum*, *Aspergillus flavus,* and *Aspergillus niger*, and *Penicillium chrysogenum* were inhibited by Romanian propolis [3]. The anticancer properties of propolis were documented by Valente et al. [4] which recorded that Portuguese propolis exhibited highly cytotoxic effect on renal cell carcinoma cells and low on normal renal cells of human. An association between anti-cancer and immunomodulatory properties of propolis was reported previously by Watanabe et al. [5]. Thailand propolis demonstrated highest anticancer activity toward five cancer cell lines with restricted activity versus normal cells [6]. Additionally, Teerasripreecha et al. [7] demonstrated that crude extracts of propolis collected from Thailand exhibited anti-proliferative effects on cancer cells. The ozonation method, recognized for its ability to prolong the stored raw vegetables and fruits shelf life [8], is currently undergoing extensive research. Ozone in current decades is used as alternative to disinfection byproducts (DBPs) in several fields. Ozone, by releasing a third oxygen atom, serves as a potent agent for eliminating microorganisms that inhabit the surfaces of vegetables and fruits. Research findings indicate that ozone has a beneficial impact on the antioxidant components, such as flavonoids and phenolic constituents [9]. Ozone can, however, degrade food flavor, discolor surface of foods, and destroy nutrients if it is administered improperly [7]. According to Sudheer et al. [10], the contents of active compounds in *Ganoderma lucidum* improved by exposure to ozone gas. In the food factories, ozonation is perhaps one of the main operative processes for sterile the newly synthesized products [11]. Guerra‐Blanco et al. [12] mentioned that Ozonated oils revealed promising findings for clinical utilization; also, the changes of structure and viscosity were depended on the ozonation degree. A recent study [13] demonstrated that ozonation of black seed oil alters the relative dose of bioactive constituents, especially thymoquinone. This study aims to evaluate the impact of ozone treatment on the phytochemical composition of propolis and to investigate its biological activities—including antimicrobial, anticancer, antioxidant, and anti-inflammatory properties—as well as its molecular interactions through docking analysis.

## Materials and methods

### Ozonation of propolis

An ozonation generator powered by electric shock created ozone gas. A 1.0 L Drechsel tank containing 0.5 mL of propolis (obtained from Sakaka, Saudi Arabia) was set up in a cooling bath at −6 °C close to the output of plasma reactor. At a flow level of 0–6 L/min, the ozone created bubbles in the propolis sample for four hours, resulting in a partially solid state. The propolis was removed from the Drechsel jar, put in an unfilled container and kept at 3 °C once it had been ozonized [13].

High-Performance Liquid Chromatography (HPLC) AnalysisThe Waters 2695 Alliance HPLC system with a UV-Vis DAD was employed to perform the HPLC analysis of the flavonoids and phenolic constituents. A Waters Sunfire TM C18 chromatography column with dimensions of 250 mm by 4.6 mm and a particle range of 5 μm was operated for the separation. An autoinjector was operated to introduce the solutions of phenolic standard and combinations into the system. To determine the best separation technique for the standards, various gradient and isocratic mobile phases were valued at diverse flow charges and column temperatures. Acetonitrile (as mobile phase A) with phosphoric acid (as mobile phase B), which was made by dropwise adding 85% orthophosphoric acid to HPLC grade water pending pH = 2, are used in the gradient method that was ultimately elected after a number of preliminary examinations. The general runtime of the approach turned into 60 min and the attention gradient turned into various as tracks: a) to start with 5 and 95% of A and B, respectively, b) 15 min, 35 and 65% of A and B, respectively, c) 20 min, 35 and 65% of A and B, respectively, d) 30 min, 40 and 60% of A and B, respectively, e) 35 min, 40 and 60% of A and B, respectively, f) forty min, 50 and 50% of A and B, respectively, g) 52 min, 70 and 30% of A and B, respectively, h) 60 min, 5 and 95% of A and B, respectively A regular glide charge of 0.5 mL/min and 5 °C have been operated. Following the evaluation of the UV-Vis spectra of the standards, 210, 280 and 360 nm of wavelengths have been selected for investigation in this experiment utilize the HPLC.

### *In vitro* antimicrobial assay

The activity of ozonized and non-ozonized propolis against microorganisms (*Bacillus subtilis* ATCC 6633, *Klebsiella pneumoniae* ATCC13883, *Salmonella typhi* ATCC 6539, *Penicillium glabrum* Op 694171, and *Candida albicans* ATCC 10221) was detected by the agar-well diffusion technique. Freshly prepared suspensions of fungi and bacteria of 100 µL were streaked onto PDA (for fungi) or Tryptic Soy Agar (for bacteria) plates. Then via a sterile cork borer, wells 0.6 cm were cut and 100 µL of extracts were introduced into the wells. The cultivated microorganisms were incubated for 48 for bacteria and 72 h for bacteria at 37 °C for bacteria and 30 °C for fungi [14]. The diameters of inhibition zones were measured. Antibiotic chloramphenicol at100 mg/mL and Gentamycin/Nystatin was applied as standard antibacterial/antifungal agent while the negative control was DMSO.

### Detection of minimal inhibitory dose (MID) and minimal bactericidal dose (MBD)

Micro-dilution broth procedure was utilized to detect the ozonized and non-ozonized propolis’ MID. The propolis extract was diluted at range varied from 3.5 to 500 µg/mL, then 200 µL of each dilution was placed in a hole of the 96-well micro-titrate plate. The bacterial and *C. albicans* suspension (2 × 10^6^ CFU/mL) with 2.0 µL of sterile NaCl (0.9%) was injected to each well. Next, it cultured at 35 °C/48h. The MID of the ozonized and non-ozonized propolis were optically measured that occurred when the reference strain growth was completely inhibited. Ozonized and non-ozonized propolis lacking an inoculum was used as a negative reference, while the inoculum without the ozonized and non-ozonized propolis used as a positive reference on the microplate. Microbial culture (100 mL) was sub-cultured onto plates containing 100% growth suppression on growth medium from the control growth, and from the inoculum without the ozonized and non-ozonized propolis. This permitted for the estimation of MBD. The MBD was recorded to possess the lowest quantity of tested samples that did not inducing growth of microorganisms during incubation time at proper condition [14].

### Propagation of cancer cell lines

The Caco-2 cells were sub-cultured in Dulbecco’s Modified Eagle Medium (DMEM)amended with fetal bovine serum (10%), and penicillin-streptomycin (1%). Cells were kept incubator with 5% CO_2_ and relative humidity (95%). The cells at level of 2 × 10^3^ cells/well were inoculated in 96-well microtiter plates for the MTT (methyl tetrazolium bromide test).

### Anti-neoplastic action of ozonized and non-ozonized propolis extracts

Via MTT assay, the anticancer effects of ozonized and non-ozonized propolis extracts were assessed in vitro against Caco-2 cells. The Caco-2 cells were seeded at a known density in 96-well microtiter plates and kept for one day at 37°C besides 5% CO_2_ and 95% relative humidity in order to improve cell attachment. Following incubation, the cells were treated with ozonized and non-ozonized propolis extracts at doses of 0, 31.25, 62.5, 125, 250, 500, and 1000 μg/mL. As a positive control, doxorubicin was employed, and the same concentrations of fresh media were applied to a blank control. After 48 hours of incubation in a CO_2_ incubator, the plates were aspirated to remove the media from each well. A fresh medium was then added after the cells had been cleaned with phosphate buffer saline (PBS) solution. Every well received a 30 µL aliquot of MTT (5 mg/mL in PBS), which was then incubated for 4 hours at 37°C. After aspirating the solution, any formazan crystals that had developed were dissolved by adding dimethyl sulfoxide (DMSO). Following a one-hour incubation period with cell-counting kit solutions, absorbance was assessed at 450 nm using a Versa Max microplate reader, and the resultant IC_50_ value was used to express the amount of cell growth inhibition by each extract [15]. The cells were examined and imagined via a microscope (Accu-Scope INC, USA) amended by a CCD camera.

### Flow cytometry for Caco-2 Apoptosis detection

Following exposure of Caco-2 cells to ozonized and non-ozonized propolis extracts, the separated cells were rinsed with PBS, reconstituted in binding buffer, and then exposed to either hypotonic propidium iodide buffer or Annexin V-FITC-PI staining in order to detect the sub-G1 peak. Following treatment, 1X binding buffer (500 μL) was used to trypsinize and resuspend Caco-2 cells. Next, Annexin-V-FITC and 5 μL of propidium iodide were added, and the mixture was left in the dark. The washed cells were resuspended in a propidium iodide solution that contained sodium citrate (0.1%) and Triton X-100 (0.1%) in order to identify the sub-G1 peak. The cells were then incubated for 30 minutes at 37°C. Lastly, flow cytometry was used to examine the cells [16].

### Antioxidant activity

Using the methodology described in Bédoui et al. [16], the ozonized and non-ozonized propolis extract’s capacity to scavenge free radical of DPPH (2,2-Diphenyl-1-picrylhydrazyl (DPPH) free radical scavenging assay radicals was assessed. First, each test extract was diluted in methanol to different doses up to 1000 μg/mL, and a 0.1 mM DPPH solution in methanol was constructed. An equal volume of DPPH solution (100 μL) and the test samples (100 μL) were then combined to create a reaction mixture, which was then allowed to sit at 30°C in the dark. Using a microplate reader, at 517 nm the absorbance was measured following a 30-minute of incubation time. All tests were performed with positive control (ascorbic acid).


$$\text{DPPH radical scavenging activity }(\%)\;=\frac{\text{Abs of control}-\text{ Abs of treatment}}{\text{Abs of control}}\times \text{100}$$ (1)

Where: Abs_control_ characterizes the DPPH radical absorbance of methanol, Abs_sample_ characterizes the absorbance of DPPH radical + treatment.

### Anti-inflammatory potential via bovine serum albumin (BSA) denaturation Assess

The anti-inflammatory potential of ozonized and non-ozonized propolis extracts was assessed. Different doses of the tested extracts comprising 1.56, 3.12, 6.25, 12.5, 25, 50, 100, and 200 µg/mL were mixed individually to 450 µL of bovine serum albumin (1% aqueous solution). Via 1N of hydrochloric acid, the solution pH was adjusted at 6.3. The mixture of reaction was preserved for 20 min at 25 °C, and then warmed for 30 min at 55°C in a water bath. The mixture of the reaction was cooled, and their absorbance was recorded at 670 nm (Biosystem 310 plus spectrophotometer). The effect of anti-inflammatory potential of ozonized and non-ozonized propolis extracts was compared with standard Diclofenac sodium. DMSO without ozonized and non-ozonized propolis extracts was employed as control [17]. The denaturation of protein (%) was estimated employing from the next equation:


$$𝐈𝐧𝐡𝐢𝐛𝐢𝐭𝐢𝐨𝐧\;𝐨𝐟\;𝐩𝐫𝐨𝐭𝐞𝐢𝐧\;𝐃𝐞𝐧𝐚𝐭𝐮𝐫𝐚𝐭𝐢𝐨𝐧\;(\%)\;=\frac{\text{Abs of control}-\text{ Abs of treatment}}{\text{Abs of control}}\times \text{100}$$ (2)

### Molecular docking study

Molecular simulation serves as vital for the rational discovery and identification of drugs techniques. Molecular docking, a stable and flexible in silico method that employs a novel computational methodology, was used to anticipate the binding behavior of each protein and choose ligands. These docking computations were performed with the Molecular Operating Environment (MOE) program. We carried out molecular docking procedures on rosmarinic acid and hespertin utilising the crystal structures of *K. pneumonia* (PDB ID: 6T77) and Caco2 cancer cell (PDB ID: 1M17). The structure of rosmarinic acid and hespertin was obtained from the PubChem website and constructed using energy minimisation and partial charge optimisation. The proteins utilised came from the RCSB website (www.rcsb.org). After the removal of any water molecules from the protein, each target has been built using corrections, 3D hydrogenations, and energy minimisation. Docking was performed on the stiff receptor atoms, and the ligand was inserted into the site employing the triangle matcher method. The scoring function was London dG, and rescoring was performed using GBVI/WSA dG procedures. Dummy atoms were made from the obtained alpha spheres. The docked ligand’s leading conformations were determined by comparing RMSD values, binding energies, and binding modes to important residues. Suppose that the procedure can discover the best position within a predetermined Root Mean Square Deviation (RMSD) value from the known conformation (often 1.5 or 2 Å, depending on ligand size). In that case, the approach is said to as “validated”.

### Statistical study

Every experiment was performed replicates, and the results are displayed as mean ±SD. After one-way investigation of variance (one-way ANOVA), which was done by Graph Pad Prism V5 (San Diego, CA, USA) software to analyze the outcomes.

## Result and discussion

The effect of ozone on chemical characterization of propolis was investigated via HPLC as illustrated in Table 1. The most of detected flavonoids and phenol compounds were recorded with high concentrations in ozonized propolis compared with non-ozonized propolis. For instance, hesperetin, acids of rosmarinic, coumaric, caffeic, and chlorogenic were induced by ozone with increasing level 92.30, 40.14, 32.03, 23.67, and 9.03%, respectively. On the other hand, five compounds namely methyl gallate, rutin, naringenin, and cinnamic acid were affected negatively by ozone where it decreased in the ozonized propolis. Other findings were visualized in Table 1. Woźniak et al. [18] reported the presence of catechin epicatechin, myricetin, pinobanksin, vanillic acid, caffeic acid and syringic acid in propolis samples, with notably high concentrations. *P-*coumaric acid cinnamyl ester, cinnamic acid phenethyl ester, *p-*coumaric acid benzyl ester, caffeic acid benzyl ester, ferulic acid benzyl ester, and caffeic acid ethyl ester were detected in Korean propolis extracted by ethanol [19]. Sudheer et al. [10] reported that ozone treatment enhanced the levels of bioactive compounds in *Ganoderma lucidum*, specifically phenolics, water-soluble polysaccharides, and flavonoids. The source of propolis also affected the biological functions of its extracts. Generally, propolis has a different and complex chemical structure with high doses of compounds associated to flavonoids and phenols existent in the extract and mostly depends on the climatic conditions, geographical region, the plants that live near, kind of bees, and the gathering time of propolis.

**Table 1:** Ozonized and non-ozonized propolis analysis by HPLC for polyphenolic and flavonoids identification.

Compound name	Non-ozonized propolis		Ozonized propolis	
	Area	Conc. (µg/g)	Area	Conc. (µg/g)
Gallic acid	93.90	343.76	116.41	426.15
Chlorogenic acid	162.50	1132.17	178.63	1244.59
Catechin	0.00	0.00	0.00	0.00
Methyl gallate	18.69	52.29	3.83	10.71
Caffeic acid	69.16	177.41	90.61	232.43
Syringic acid	59.37	174.60	60.81	178.85
Rutin	15.99	119.65	10.92	81.75
Coumaric acid	268.77	482.98	395.42	710.56
Ellagic acid	0.00	0.00	0.00	0.00
Vanillin	12.81	23.23	14.65	26.57
Ferulic acid	28.27	78.04	26.04	79.58
Naringenin	432.78	1997.16	402.06	1855.37
Rosmarinic acid	616.41	2993.26	1029.70	5000.18
Daidzein	483.04	1382.89	484.29	1386.47
Querectin	3.21	19.99	6.67	41.53
Cinnamic acid	846.43	820.24	776.91	752.87
Kaempferol	98.36	123.64	149.26	187.61
Hesperetin	169.43	396.68	2201.08	5153.38

**Fig 1:**
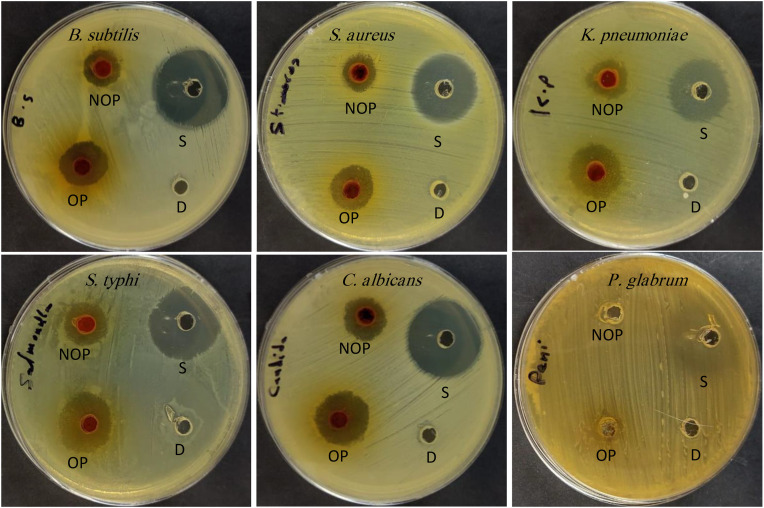
Antimicrobial activity of ozonized propolis (OP), non-ozonized propolis (NOP) extracts, standard (Gentamycin/Nystatin), and DMSO (D) negative control.

Propolis extracts in the present investigation reflected suppression effect against different microorganisms; however, their effect was less than caused by Gentamycin/Nystatin (Table 2 and Fig 1). The investigated bacteria and *C. albicans* were more susceptible to ozonized propolis than non-ozonized propolis indicating that ozone alters the composition of propolis and therefore their effects were clear against tested microorganisms. For example, the inhibition area was 20 ± 1 and 22 ± 2 mm using ozonized propolis while it was 12 ± 1 and 14 ± 1 mm using non-ozonized propolis *against K. pneumoniae* and *S. typhi,* respectively. The filamentous fungus *P. glabrum* was not suppressed by either non-ozonized propolis or ozonized propolis. This perhaps may be due to the differences in cell wall constituents. Moreover, the phytochemical constituents of propolis may play a crucial role in inhibiting microbial growth. The results in Table 2 also indicate that the MID of non-ozonized propolis was lower than that of ozonized propolis. Different extracts of Argentinian propolis inhibited numerous yeasts and dermatophytes fungi with MID values ranged from 16 and 125 μg/mL, moreover 2′,4′-dihydroxychalcone and 2′,4′-dihydroxy-3-methoxychalcone were the main bioactive constituents in the extracts which give MID values ranged from 1.9 and 2.9 μg/mL) [2]. Killing kinetic time assay visualized the efficacy of ozonized propolis against tested bacteria compared to non-ozonized propolis. The colony-forming units (CFU) of all tested bacteria decreased with increasing killing kinetic time, particularly when treated with ozonized propolis. At 150 min *S. aureus* was completely inhibited, while at 180 min all tested bacteria were inhibited using ozonized propolis (Table 3). Ethanolic extract of Malaysian propolis presented the antibacterial activity toward *B. subtilis*, *S. aureus*, *Enterococcus faecalis*, *Micrococcus luteus*, *Enterococcus faecium*, and *Streptococcus mutans* [20].

**Table 2:** MID, MBD and antimicrobial activity of ozonized propolis and non-ozonized propolis.

Tested microorganisms	Inhibition zones (mm)			MID		MBD	
	Non-ozonized propolis	Ozonized propolis	Positive control (Gentamycin/Nystatin)	Non-ozonized propolis	Ozonized propolis	Non-ozonized propolis	Ozonized propolis
*B. subtilis*	13 ± 1^a^	17 ± 1^b^	27 ± 1^c^	125	62.5	250	125
*S. aureus*	12 ± 1^a^	16 ± 1^b^	24 ± 1^c^	125	31.25	250	62.5
*K. pneumoniae*	12 ± 1^a^	20 ± 1^b^	23 ± 1^c^	250	31.25	500	31.25
*S. typhi*	14 ± 1^a^	22 ± 2^b^	24 ± 1^c^	125	15.62	500	31.25
*C. albicans*	14 ± 2^a^	19 ± 1^b^	27 ± 1^c^	125	31.25	250	62.5
*P. glabrum*	NA	NA	28 ± 1	--	--	--	--

Values are shown as mean ± SD, with different letters denoting significant differences (P ≤ 0.05) at each row.

**Table 3:** Effect of different Killing Kinetic times of ozonized propolis and non-ozonized propolis on various bacteria.

Killing Kinetic time (min)	CFU							
	Non-ozonized propolis				Ozonized propolis			
	*B. subtilis*	*S. aureus*	*K. pneumoniae*	*S. typhi*	*B. subtilis*	*S. aureus*	*K. pneumoniae*	*S. typhi*
**0**	27 × 10^5a^	32 × 10^5a^	76 × 10^5a^	171 × 10^5a^	27 × 10^5a^	32 × 10^5a^	76 × 10^5a^	171 × 10^5a^
**30**	272 × 10^4b^	182 × 10^4b^	228 × 10^4b^	23 × 10^4b^	282 × 10^3b^	212 × 10^b^	232 × 10^4b^	158 × 10^3b^
**60**	14 × 10^4c^	218 × 10^3c^	180 × 10^3c^	25 × 10^3c^	157 × 10^2c^	165 × 10^2c^	175 × 10^2c^	127 × 10^2c^
**120**	212 × 10^2d^	205 × 10^d^	134 × 10^2d^	18 × 10^2d^	122^d^	105^d^	68 × 10^d^	256^d^
**150**	15 × 10^e^	114^e^	295^e^	175^e^	14^e^	0	12^e^	18^e^
**180**	0	0	23^f^	11^f^	0	0	0	0

Values are shown with different letters denoting significant differences (P ≤ 0.05) at each column.

**Fig 2:**
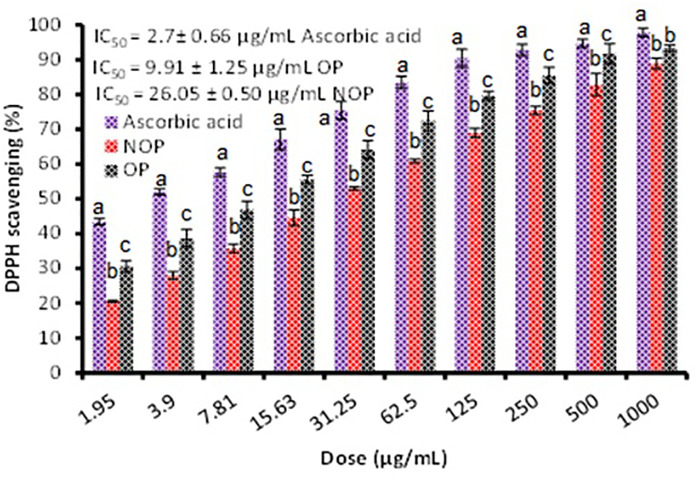
Effect of ozonized and non-ozonized propolis extracts on DPPH scavenging. Values are shown as mean ± SD (bars), with different letters denoting significant differences (P ≤ 0.05) at each concentration.

From Fig 2, it is evident that the activity of ozonized and non-ozonized propolis extracts exhibited excellent antioxidant potency indicated by an IC_50_ quantity of 9.91 ± 1.25 and 26.05 ± 0.50 μg/mL, correspondingly. Moreover, from the obtained results ozonized propolis extract was stronger than non-ozonized propolis extracts for DPPH scavenging at all tested doses. This remark suggests a limited existence of active constituent’s concentration within the non-ozonized propolis extracts able to neutralizing DPPH radicals. Otherwise, the remarked antioxidant potential may be affected by the number of active compounds. According to Kustiawan et al. [21], Indonesian propolis exhibited antioxidant activity, as measured by the DPPH assay, with an IC₅₀ value of 50.61 μg/mL. Zohdi et al. [22] studied the antioxidant via DPPH of collected propolis by honeybees (*Apis dorsata*) and stingless bees (*Heterotrigona itama*) from various areas in Malaysia with IC_50_ quantity of 85.02 ± 1.59 and 61.22 ± 1.48 μg/mL, respectively.

**Fig 3:**
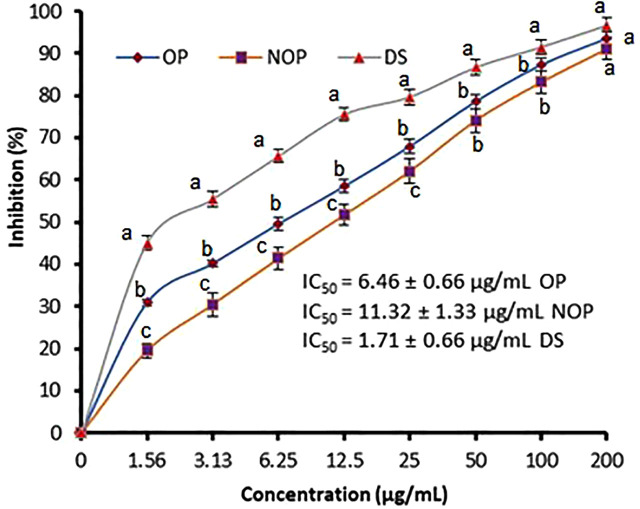
Anti-inflammatory potential of ozonized and non-ozonized propolis extracts via inhibition of protein denaturation. Values are shown as mean ± SD (bars), with different letters denoting significant differences (P ≤ 0.05) at each concentration.

The inhibition of protein denaturation for measuring the anti-inflammatory ability of ozonized and non-ozonized propolis extracts was illustrated in Fig 3. Inhibition (%) of protein denaturation increased with increasing the dose of propolis extracts with dose dependent manner up to 200 µg/mL. The inhibition result of protein denaturation was excellent using the ozonized propolis extract (IC_50_, 6.46 ± 0.66 μg/mL), while the IC_50_ value was 11.32 ± 1.33 μg/mL using non-ozonized propolis. The anti-inflammatory and antioxidant properties of propolis were described in a study by Oršolić [23], which linked these activities to the presence of caffeic acid, quercetin, and galangin in the propolis extract. Inhibition of inflammation markers such as myeloperoxidase, ornithine decarboxylase, hyaluronidase, and tyrosine-protein-kinase was associated with these compounds. The mechanism of propolis for management of inflammation was studied also by Kustiawan et al. [24], where decline of COX-2 and preventing ROS in the synthesis of arachidonic acid. Some of the detected flavonoids such as rosmarinic acid, naringenin, and hesperetin in propolis extract of our study and other investigations succeeded to prevent the inflammation via control the of inflammation-related genes expression namely TNF-α, iNOS, and IL-1β [25,26].

**Fig 4:**
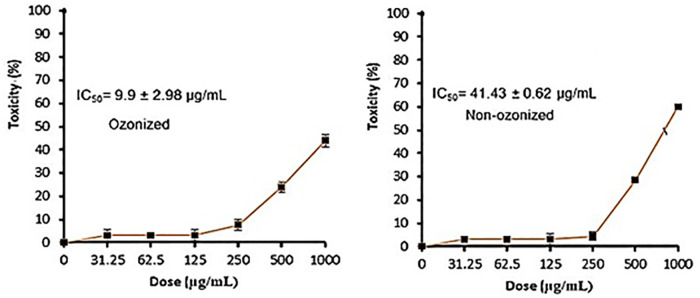
Toxicity of propolis extract against Caco-2 cell lines.

**Fig 5:**
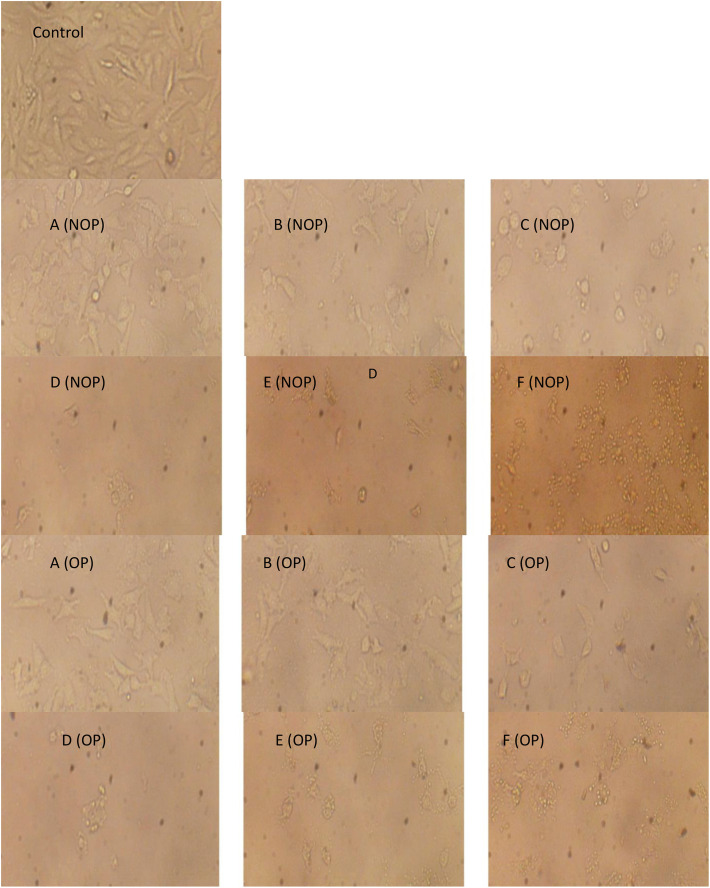
Morphological characteristics of Caco cancer cell lines following treatment with varying concentrations of ozonized (OP) and non-ozonized (NOP) propolis extracts. A (31.25 µg/mL), B (62.5 µg/mL), C (125 µg/mL), D (250 µg/mL), E (500 µg/mL), and F (1000 µg/mL).

**Fig 6:**
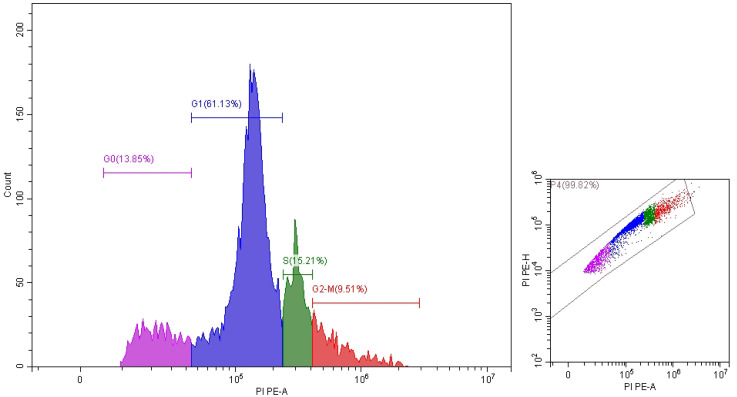
Cell cycle of control Caco-2.

**Fig 7:**
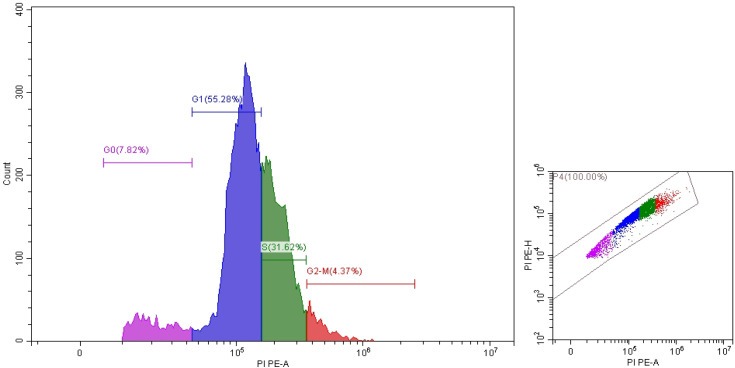
Cell cycle of Caco-2 treated by NOP.

**Fig 8:**
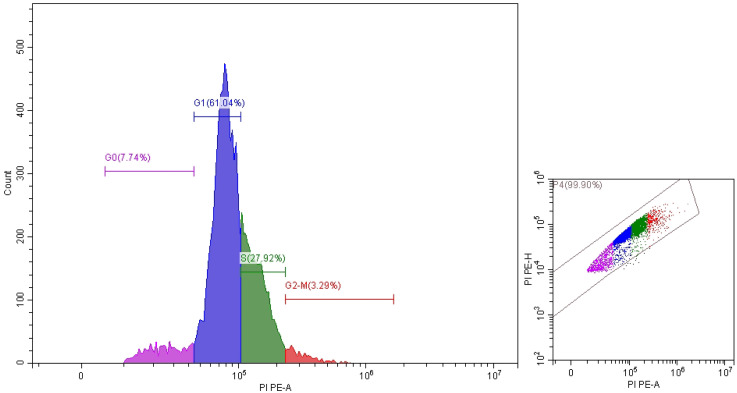
Cell cycle of Caco-2 treated by OP.

It is clear that propolis exhibits moderate anticancer properties, but ozonation process increased their anticancer activity. These results were documented via IC_50_ values which were 41.43 ± 0.62 and 9.9 ± 2.98 μg/mL utilized non-ozonized and ozonized propolis, respectively (Fig 4). Both ozonized and non-ozonized propolis extracts exhibited dose-dependent toxicity toward Caco-2 cell lines. From the present experiment, critical role was associated with the ozone for induce the anticancer potential of propolis. However as mentioned in the introduction, the biological activities of propolis may depend on its origin, chemical constituents, extraction solvents, tested cells. Propolis extract exhibited different IC_50_ values 298, 185.6, 250.7, and 292.9 μg/mL against different cancer cells SW-620, DU-145, PC-3, and MCF-7, respectively [27]. IC_50_ value of Portuguese propolis was 56.1 μg/mL against renal cell carcinoma cells (Valente et al. 2011). Thailand propolis provides IC_50_ values ranged from 4.09–14.7 μg/mL versus cancer cells [6]. In another study, the extracted Thailand propolis via hexane and dichloromethane demonstrated IC_50_ values (41.3 to 52.4 μg/mL and 43.8 to 53.5 μg/mL) versus five cancer cell lines, correspondingly, moreover cardanol and cardol were main bioactive constituents in the extracts which give IC_50_ values 10.8 to 29.3 μg/mL against the same cell lines [6]. According to Durmaz et al. [27] the action mechanism of propolis extract against cancer cells are still obscure, although numerous investigations recorded its anticancer effect against several kinds of cancer, who indicated that levels of PPP2R1A (Protein Phosphatase 2 Scaffold Subunit Aalpha) and markers of apoptosis in cancer cell lines were induced by propolis. Changes in Caco-2 cell morphology were observed following exposure to non-ozonized and ozonized propolis, with cells shifting from a spindle-shaped to a rounded form, particularly at higher doses of ozonized propolis. Moreover, aggregation of cells was observed at 500 and 1000 μg/mL of both non-ozonized and ozonized propolis (Fig 5). Employing annexin V-FITC and propidium iodide staining, flow cytometric analysis for arrest of Caco-2 cells cycle and apoptosis revealed that propolis could cause dose-dependent apoptosis and cycle arrest in the G0/G1 stage (Figs 6–8). These findings imply that propolis in the current study may be a potential substitute agent for the inhibition and treatment of Caco-2 cells.

**Fig 9:**
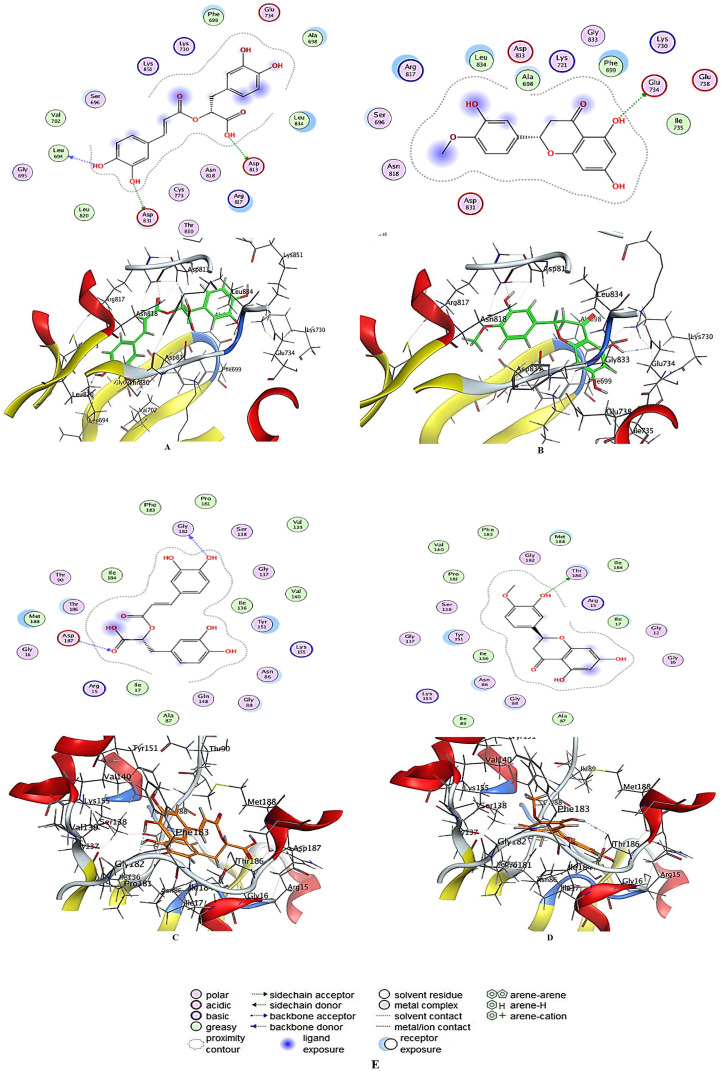
2D and 3D diagrams viewing the interaction between: rosmarinic acid and active sites of Caco2 cancer cell 1M17 protein. (A), hespertin and active sites of Caco2 cancer cell 1M17 protein (B), rosmarinic acid and active sites of *K. pneumoniae* 6T77 protein (C), hespertin and active sites of *K. pneumoniae* 6T77 protein (D), and the illustrative key for the kinds of interaction among ligands and selected protein receptors (E).

Molecular docking analyses the interactions and binding affinities of studied compounds (rosmarinic acid and hespertin) as therapeutic molecules, providing information into their effectiveness against the target. The docked complex conformations were investigated based on docking scores and ligand interactions with *K. pneumonia* (PDB ID: 6T77) and Caco2 cancer cell (PDB ID: 1M17) proteins, which were elected based on the literature and prior investigations. The docking scores and binding energies of rosmarinic acid and hespertin with the *K. pneumoniae* protein structure (PDB ID: 6T77) are summarized in Table 4. Rosmarinic acid exhibited docking scores ranging from −6.47481 to −6.9092, with its most favorable binding energy of −6.9092 kcal/mol and associated RMSD_refine values of 1.11 to 3.14. The highest E_place and E_refine values were observed at −113.6 and −39.692, respectively, indicating strong binding potential. Interaction analysis revealed hydrogen bonding with residues such as GLY 182 and ASP 187, with bond distances of 2.86 Å and 3.33 Å, correspondingly. Hespertin showed docking scores ranging from −6.17663 to −6.44174, with a favorable binding energy of −6.44174 kcal/mol and RMSD_refine values between 1.00 and 1.93. Key interactions included hydrogen bonding with THR 186, having a bond distance of 2.97 Å and binding energy of −1.3 kcal/mol. On the other hand, rosmarinic acid docking scores against the Caco2 cancer cell protein (PDB ID: 1M17) varied from −6.505 to −6.91777 kcal/mol as shown in Table 5. The most optimal conformation has a binding energy of −6.91777 kcal/mol, with E_place and E_refine values of −94.4898 and −41.4202, respectively. Hydrogen bonding interactions were detected with ASP 813, LEU 694, and ASP 831, with bond lengths of 2.76, 2.68, and 2.75 Å, involving binding energies ranging from −1.2 to −8.6 kcal/mol. Where hespertin had docking scores reaching from −5.90455 to −6.36651 kcal/mol, with the maximum binding energy of −6.36651 kcal/mol, and E_place and E_refine values of −70.1577 and −32.303, respectively. GLU 734 exhibited a significant hydrogen bond interaction with a binding energy of −0.8 kcal/mol and a bond distance of 2.95 Å. All the interactions for the two targets are illustrated in Tables 6, 7. The molecular docking documents highlight the potential of rosmarinic acid and hespertin as promising candidates against both bacterial and cancer targets. For *K. pneumoniae* (PDB ID: 6T77), rosmarinic acid demonstrated stronger docking scores and binding affinities compared to hespertin. This superior binding can be attributed to its ability to form stable hydrogen bonds with critical active site residues such as GLY 182 and ASP 187. Additionally, the higher E_place and E_refine values for rosmarinic acid indicate a more stable binding conformation within the active site. Hespertin, while slightly less effective, still displayed significant interactions with residues like THR 186, reinforcing its potential as an antimicrobial agent. Similarly, against the Caco2 cancer cell protein (PDB ID: 1M17), rosmarinic acid exhibited superior binding affinities compared to hespertin. Notably, the interaction of rosmarinic acid with ASP 813 (binding energy of −8.6 kcal/mol) underscores its strong binding potential and suggests a pivotal role in disrupting the active site functionality. Hespertin, despite lower docking scores, demonstrated interactions with residues such as GLU 734, further supporting its role as a complementary therapeutic candidate. Optimal configuration of rosmarinic acid and hespertin within the investigated proteins’ core activity was illustrated in Fig 9A–D. Numerous investigators employed molecular docking methodology to link among any developed or discovered substance and target construction to assess biological actions of these substances [28–34]. Moreover, Fig 9E showed the key for the kinds of interaction among ligands and selected protein receptors. Despite the promising findings presented, some limitations should be acknowledged. Firstly, the study was conducted *in vitro*, and the observed biological activities of ozonized propolis (OP) may not directly involved *in vivo* outcomes. The complex interaction between propolis compounds and physiological systems requires further pharmacokinetic and pharmacodynamic evaluations. Secondly, although the study identified and quantified major phytochemicals using HPLC, a more comprehensive metabolomic profiling using LC-MS/MS or NMR could provide a deeper understanding of the chemical alterations due to ozonation. Future studies should expand to include other cancer cell lines and clinically relevant pathogens.

**Table 4:** Docking scores and energies of rosmarinic acid and hespertin with Caco2 cancer cell (PDB ID: 1M17).

Mol	S	rmsd_refine	E_conf	E_place	E_score1	E_refine	E_score2
Rosmarinic acid	−6.91777	1.4808475	−37.4107	−94.4898	−14.2351	−41.4202	−6.91777
Rosmarinic acid	−6.6202	1.5713278	−38.9231	−81.1586	−13.3348	−36.8325	−6.6202
Rosmarinic acid	−6.59157	1.3921747	−37.2143	−84.0199	−16.7625	−35.0971	−6.59157
Rosmarinic acid	−6.56541	3.0471804	−27.9498	−85.6631	−13.6525	−38.4251	−6.56541
Rosmarinic acid	−6.505	1.1691413	−41.055	−86.3564	−13.9409	−38.5207	−6.505
Hespertin	−6.36651	1.342549	−23.8741	−70.1577	−11.8173	−32.303	−6.36651
Hespertin	−6.30354	3.1665442	−27.4136	−97.6554	−11.7223	−35.538	−6.30354
Hespertin	−6.21285	2.9119906	−23.8394	−106.227	−12.0431	−34.9245	−6.21285
Hespertin	−6.0409	1.0116593	−23.6425	−80.2692	−12.3992	−33.0334	−6.0409
Hespertin	−5.90455	0.93938071	−25.7505	−77.0555	−11.9734	−32.004	−5.90455

**Table 5:** Interaction of rosmarinic acid and hespertin with Caco2 cancer cell (PDB ID: 1M17).

Mol	Ligand	Receptor	Interaction	Distance	E (kcal/mol)
Rosmarinic acid	O 17	OD2 ASP 813 (A)	H-donor	2.76	−8.6
	O 35	O LEU 694 (A)	H-donor	2.68	−1.2
	O 37	OD2 ASP 831 (A)	H-donor	2.75	−2.4
Hespertin	O 33	OE1 GLU 734 (A)	H-donor	2.95	−0.8

**Table 6:** Scores of dockings and energies of rosmarinic acid and hespertin with K. pneumoniae (PDB ID: 6T77).

Mol	S	rmsd_refine	E_conf	E_place	E_score1	E_refine	E_score2
Rosmarinic acid	−6.9092	2.137475	−44.9081	−83.9006	−13.1725	−36.9445	−6.9092
Rosmarinic acid	−6.78135	3.143722	−37.6726	−91.1353	−13.6766	−39.692	−6.78135
Rosmarinic acid	−6.69059	1.160592	−38.7887	−113.6	−14.7324	−39.4894	−6.69059
Rosmarinic acid	−6.63295	1.173487	−36.6714	−104.008	−14.8461	−39.4427	−6.63295
Rosmarinic acid	−6.47481	1.106244	−28.772	−107.619	−13.5338	−31.9363	−6.47481
Hespertin	−6.44174	1.004136	−20.1869	−77.0783	−13.3467	−34.4305	−6.44174
Hespertin	−6.36953	1.840313	−23.6025	−71.7749	−12.9267	−33.8891	−6.36953
Hespertin	−6.24113	1.472228	−26.3667	−82.3968	−12.8456	−33.8298	−6.24113
Hespertin	−6.18634	1.938379	−25.534	−78.5957	−12.3026	−34.1365	−6.18634
Hespertin	−6.17663	1.826065	−27.8031	−114.41	−13.3318	−35.3093	−6.17663

**Table 7:** Interaction of rosmarinic acid and hespertin with K. pneumoniae (PDB ID: 6T77).

Mol	Ligand	Receptor	Interaction	Distance	E (kcal/mol)
Rosmarinic acid	O 35	O GLY 182 (A)	H-donor	2.86	−2.0
	O 16	N ASP 187 (A)	H-acceptor	3.33	−0.6
Hespertin	O 35	OG1 THR 186 (A)	H-donor	2.97	−1.3

## Conclusion

Several compounds were detected in OP at high concentration, suggesting that ozone treatment enhances the release of compounds from the propolis extract. This effect was associated with increased antimicrobial, antioxidant, anti-inflammatory, and anticancer potential of OP. The molecular docking studies underscore rosmarinic acid as a highly promising dual-target agent against both *K. pneumoniae* (PDB: 6T77) and Caco2 cancer cell proteins (PDB: 1M17). Hespertin, while less potent, demonstrates complementary therapeutic potential through interactions with key residues (THR 186 in bacterial targets; GLU 734 in cancer targets), suggesting its utility as a secondary or adjunct agent. These findings highlight the therapeutic potential of both compounds in the development of antimicrobial and anticancer drugs.

## Supporting information

S1 Data Raw data. (RAR)
